# Long-term prognosis of diabetic patients with acute myocardial infarction in the era of acute revascularization

**DOI:** 10.1186/1475-2840-9-1

**Published:** 2010-01-04

**Authors:** Ayako Takara, Hiroshi Ogawa, Yasuhiro Endoh, Fumiaki Mori, Jun-ichi Yamaguchi, Atsushi Takagi, Ryo Koyanagi, Tsuyoshi Shiga, Hiroshi Kasanuki, Nobuhisa Hagiwara

**Affiliations:** 1 Division of Cardiology, Saiseikai Kurihashi Hospital, Saitama, Japan; 2 Department of Cardiology, the Heart Institute of Japan, Tokyo Women's Medical University, Tokyo, Japan

## Abstract

**Background:**

The long-term prognosis of diabetic patients with acute myocardial infarction (AMI) treated by acute revascularization is uncertain, and the optimal pharmacotherapy for such cases has not been fully evaluated.

**Methods:**

To elucidate the long-term prognosis and prognostic factors in diabetic patients with AMI, a prospective, cohort study involving 3021 consecutive AMI patients was conducted. All patients discharged alive from hospital were followed to monitor their prognosis every year. The primary endpoint of the study was all-cause mortality, and the secondary endpoint was the occurrence of major cardiovascular events. To elucidate the effect of various factors on the long-term prognosis of AMI patients with diabetes, the patients were divided into two groups matched by propensity scores and analyzed retrospectively.

**Results:**

Diabetes was diagnosed in 1102 patients (36.5%). During the index hospitalization, coronary angioplasty and coronary thrombolysis were performed in 58.1% and 16.3% of patients, respectively. In-hospital mortality of diabetic patients with AMI was comparable to that of non-diabetic AMI patients (9.2% and 9.3%, respectively). In total, 2736 patients (90.6%) were discharged alive and followed for a median of 4.2 years (follow-up rate, 96.0%). The long-term survival rate was worse in the diabetic group than in the non-diabetic group, but not significantly different (hazard ratio, 1.20 [0.97-1.49], p = 0.09). On the other hand, AMI patients with diabetes showed a significantly higher incidence of cardiovascular events than the non-diabetic group (1.40 [1.20-1.64], p < 0.0001). Multivariate analysis revealed that three factors were significantly associated with favorable late outcomes in diabetic AMI patients: acute revascularization (HR, 0.62); prescribing aspirin (HR, 0.27); and prescribing renin-angiotensin system (RAS) inhibitors (HR, 0.53). There was no significant correlation between late outcome and prescription of beta-blockers (HR, 0.97) or calcium channel blockers (HR, 1.27). Although standard Japanese-approved doses of statins were associated with favorable outcome in AMI patients with diabetes, this was not statistically significant (0.67 [0.39-1.06], p = 0.11).

**Conclusions:**

Although diabetic patients with AMI have more frequent adverse events than non-diabetic patients with AMI, the present results suggest that acute revascularization and standard therapy with aspirin and RAS inhibitors may improve their prognosis.

## Background

Treatment of coronary artery disease (CAD) has progressed rapidly since the introduction of coronary artery bypass grafting (CABG) and percutaneous coronary intervention (PCI). The introduction of antiplatelet agents, angiotensin-converting enzyme inhibitors (ACEIs), and statins has also led to marked changes in the medical management of CAD. Several large-scale clinical studies have been conducted to verify the efficacy of these therapies, and guidelines for the treatment of CAD have been established by medical societies based on the results of these studies[1]. Previous studies[2,3] clearly demonstrated that diabetic patients with CAD have a poor prognosis. However, the long-term prognosis of diabetic patients with acute myocardial infarction (AMI) is uncertain, and optimal pharmacotherapy has not been established in the contemporary acute revascularization era.

To assess the current management of AMI in Japan and the prognosis of Japanese patients, we conducted a prospective cohort study (The Heart Institute of Japan, Acute Myocardial Infarction registry: HIJAMI), in which consecutive patients with AMI who were admitted to the Department of Cardiology at The Heart Institute of Japan (Tokyo Women's Medical University) and related institutions were enrolled and followed [4]. Of the patients enrolled in the HIJAMI registry, those with diabetes mellitus were selected for the present, prospective, observational study designed to assess the clinical status of such patients, therapeutic modalities, and their prognosis, in order to determine the optimal therapeutic management of diabetic patients with AMI.

## Methods

### Study sample

Full details of the HIJAMI registry have been described previously[4]. In brief, HIJAMI is a multicenter, prospective cohort of consecutive patients with AMI who were admitted within 48 hours after the onset of symptoms. Between January 1999 and June 2001, 3021 consecutive patients from 17 participating hospitals in Japan were registered. As HIJAMI was meant for observational purposes, treatment strategies, such as drug therapies and early reperfusion treatment, were used at the discretion of the physician responsible at each hospital. Clinical and angiographic data, including the patients' demographics, coronary risk factors, therapeutic modalities, complications, number of diseased vessels, infarct-related arteries, PCI strategies, laboratory data, and outcomes were prospectively collected using a standardized case report form. AMI was diagnosed on the basis of the following criteria: (1) typical chest pain; (2) a greater than two-fold elevation of cardiac muscle enzyme levels compared to normal levels; and (3) new appearance of abnormal Q waves, an elevation or reduction of ST segments, a typical change in T waves, or new appearance of left bundle branch block[5]. When at least two of the above three criteria were met, AMI was diagnosed. All patients discharged alive from hospital were followed to monitor their prognosis every year (in December) by review of their medical records or telephone survey. The study was conducted according to the Declaration of Helsinki and was approved by the ethical review board of each institution. At the time of enrollment, informed consent for participation in the study was obtained either in writing or orally from all subjects.

Diabetes mellitus was diagnosed according to the following criteria[6]: a prior diagnosis of diabetes in a patient receiving antidiabetic treatment, or a fasting blood glucose level ≥ 126 mg/dl, a blood glucose level higher than 200 mg/dL 2 hours after the 75 g OGTT, or a random blood glucose level ≥ 200 mg/dl on two or more occasions, or these standard values were exceeded once and any of the following criteria were met (i: typical symptoms (dry mouth, polydipsia, polyuria, and weight loss), ii: HbA_1c _≥ 6.5%, or iii: presence of diabetic retinopathy). Hypertension was diagnosed when the patient had a history of hypertension or had a systolic blood pressure ≥ 140 mmHg or a diastolic blood pressure ≥ 90 mmHg during index hospitalization. Hypercholesterolemia was diagnosed when the patient had a history of dyslipidemia or a fasting total cholesterol level ≥ 220 mg/dl or a fasting triglyceride level ≥ 150 mg/dl.

### Follow-up

The primary endpoint of the study was the all-cause mortality rate, and the secondary endpoint was the occurrence of major cardiovascular events (including death from cardiovascular causes, recurrent myocardial infarction, unstable angina or heart failure requiring hospitalization, and coronary revascularization).

Two studies were performed. First, the long-term prognosis after myocardial infarction was compared between diabetic and non-diabetic patients. The patients were classified into a diabetic group with AMI and a non-diabetic group with AMI, and the primary and secondary endpoints were compared over the long term. Second, the effects of various therapies on life expectancy after myocardial infarction were assessed. To study the therapeutic modalities that affected life expectancy in diabetic patients with AMI, PCI in the acute phase and the drugs prescribed at discharge (aspirin, beta-blockers, calcium antagonists, nitrates, ACEIs, angiotensin receptor agonists (ARBs), and statins) were evaluated in diabetic patients with AMI. To balance observed covariates between the two groups, the propensity score method was used[7].

This study was funded by the Japan Research Promotion Society for Cardiovascular Diseases, which played no role in the conduct of the study.

### Statistics

Continuous variables showing a normal distribution are expressed as the mean and standard deviation. Data without a normal distribution are expressed as the median and interquartile range (IQR) (25%, 75%). Comparisons between the two groups were done using Student's *t*-test for data assumed to have a normal distribution, the Mann-Whitney U-test for data without a normal distribution or for ordered, categorical data, and the chi-square test for non-ordered, categorical data. All statistical tests were two-sided, and the level of significance was set at p = 0.05. Since this was a prospective observational study and not a comparative study using randomization, even the diabetic patients with AMI showed some differences in background factors. Therefore, the hazard ratio (HR) and its 95% confidence interval (CI) were calculated using multiple logistic regression after propensity scores were used to match subjects for the 20 factors mentioned below. Diabetic patients with AMI were divided into two groups according to use or non-use of each therapy, and patients with matched propensity scores were extracted to examine associations with life expectancy. For the calculation of propensity scores, age, sex, severity of heart failure at admission, history of hypertension, history of hypercholesterolemia, smoking habits, history of myocardial infarction, PCI at admission, left ventricular ejection fraction, number of stenosed coronary vessels, time from onset of AMI to revascularization, HbA_1c_, C-reactive protein, serum creatinine, and medications prescribed at the time of discharge from hospital (aspirin, ACEIs, or angiotensin receptor blockers (ARBs), calcium antagonists, nitrates, and statins) were used, and then propensity scores were calculated using a multiple logistic regression model[7,8]. The cumulative probabilities of event curves were estimated using the Kaplan-Meier method. To evaluate the effect of therapeutic modalities with respect to subsequent events, conventional Cox proportional hazards models were used. Statistical analysis was performed with SAS software version 9.1 (SAS Institute, Cary, NC, USA).

## Results

### Baseline characteristics

A total of 3021 consecutive patients with AMI was enrolled between January 1999 and June 2001. There were 2136 men (70.7%) and 885 women (29.3%); their average age was 69 years. Of these patients, 1102 (36.5%) had diabetes mellitus. Their average age was 66 years, 73% of them were men, and they more often had a history of hypertension, hypercholesterolemia, or myocardial infarction than the non-diabetic patients. Also, congestive heart failure was more common in the acute phase, and a higher percentage of patients had a reduced left ventricular ejection fraction and multi-vessel coronary artery disease in the chronic phase. There were no significant differences between the diabetic and non-diabetic patients with respect to CRP and renal function. During the index hospitalization, successful revascularization was achieved in 71.5% of the AMI patients with diabetes and in 77.5% of the AMI patients without diabetes. The overall death rate was 9.3% for the diabetic group and 9.2% for the non-diabetic group during index hospitalization (Table 1).

**Table 1:** Interventional Methods and In-hospital Mortality

	PCI n (%)	CT n (%)	CABG n (%)	Mortality (%)
Diabetic	612 (55.5)	168 (15.2)	9 (0.8)	9.3
Nondiabetic	1140 (59.7)	322 (16.9)	18 (0.9)	9.2

CABG: Coronary artery bypass grafting. CT: Coronary thrombolysis. PCI: Percutaneous coronary intervention.

### Long-term follow-up

A total of 2,736 patients was discharged alive and followed regularly. The average duration of follow-up for assessment of the prognosis was 4.2 years (0.0027 to 6.4 years), and the follow-up rate was 96%. In the HIJAMI study, the follow-up assessment was conducted by a telephone survey in 31.8% (with a response rate of 94.3%).

**Figure 1:**
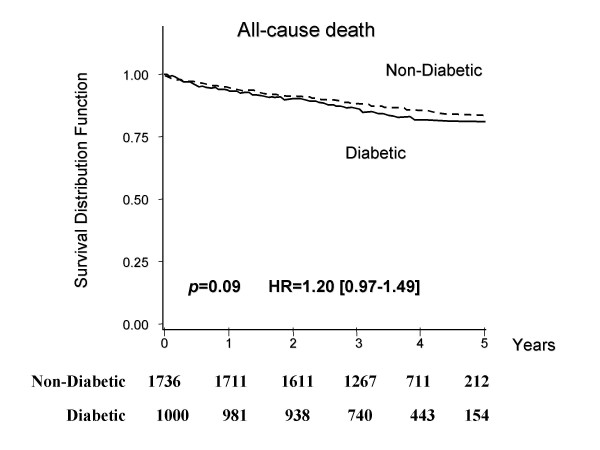
**Kaplan-Meier survival curves for all-cause mortality among AMI patients with or without diabetes**. The solid line indicates AMI patients with diabetes; the dotted line indicates those without diabetes.

**Figure 2:**
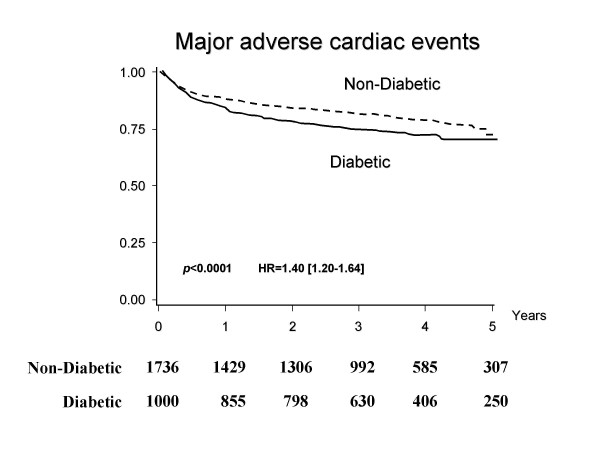
**Kaplan-Meier curves for the time until the first occurrence of a major adverse cardiovascular event (MACE), consisting of death from cardiovascular causes, recurrent myocardial infarction, angina pectoris or heart failure requiring hospitalization, and coronary revascularization, among AMI patients with or without diabetes**. The solid line indicates AMI patients with diabetes; the dotted line indicates those without diabetes

Of the patients who were discharged alive, 1,000 were diabetic (Table 2). Although the long-term survival rate was worse in the diabetic group than in the non-diabetic group, the difference in rates was not significant (HR = 1.20, 95% C. I.:0.97-1.49, p = 0.09, Figure 1). The causes of death in the diabetic group were cardiac (7.2%), including heart failure (3.0%), myocardial infarction (2.1%), and other cardiac deaths (2.1%), cerebrovascular disease (1.2%), malignancy (1.8%), and other conditions (5.2%). In the non-diabetic group, the causes of death were cardiac (4.6%), including heart failure (2.0%), myocardial infarction (0.8%), and other cardiac deaths (1.8%), cerebrovascular disease (0.9%), malignancy (1.8%), and other conditions (4.3%). The diabetic group showed a significantly higher incidence of cardiovascular events than the non-diabetic group (1.40 [1.20-1.64], p < 0.0001, Figure 2).

**Table 2:** Patient characteristics

	Non-Diabetic (N = 1,736)	Diabetic (N = 1,000)	*P*--Value
Age (years)	67 ± 13	66 ± 11	0.004
Men	1236 (71%)	726 (73%)	0.433
Killip class (%)			
I	1530 (88%)	791 (79%)	< 0.001
II	106 (6%)	85 (9%)	
III	67 (4%)	85 (9%)	
IV	33 (2%)	39 (4%)	
Hypertension	901 (52%)	591 (59%)	< 0.001
Hyperlipidemia*	624 (36%)	472 (47%)	< 0.001
Smoking	944 (54%)	562 (56%)	0.356
Prior myocardial infarction	226 (13%)	188 (19%)	< 0.001
Prior percutaneous coronary intervention	131 (8%)	98 (10%)	0.040
Left ventricular ejection fraction (%)	54 ± 13	51 ± 13	< 0.001
Number of diseased vessels			
≤ 1	846 (49%)	390 (39%)	<0.001
≥ 2	481 (28%)	322 (32%)	
Undefined	409 (24%)	288 (29%)	
Onset to reperfusion (hrs)**	4.5 [3.1-7.0]	4.6 [3.1-7.8]	0.881
HbA1c (%)	5.3 ± 0.5	7.1 ± 1.6	<0.001
C-reactive protein (mg/dl)**	0.3 [0.1-0.7]	0.3 [0.1-0.9]	0.027
Serum creatinine (mg/dl)**	0.8 [0.7-1.0]	0.9 [0.7-1.1]	0.048
Concomitant medications			
Renin-angiotensin system inhibitors	1095 (63%)	634 (63%)	0.866
β-blockers	522 (30%)	358 (36%)	0.002
Calcium antagonists	489 (28%)	356 (36%)	< 0.001
Nitrates	1008 (58%)	669 (67%)	< 0.001
Aspirin	1550 (89%)	881 (88%)	0.343

Plus minus values are mean ± S.D. *:Hyperlipidemia was defined as total cholesterol > 220 mg/dl. **:Values are median [interquartile range]

### Prognostic factors associated with long-term survival

Aspirin was prescribed at discharge to 1,550 (89%) of the 1,736 non-diabetic AMI patients, and 881 (88%) of the 1,000 diabetic AMI patients. ACEIs and ARBs were also prescribed at relatively high rates (63%) in both groups. However, β-blockers, calcium antagonists, and nitrates were prescribed at higher rates in the diabetic group (Table 2).

**Figure 3:**
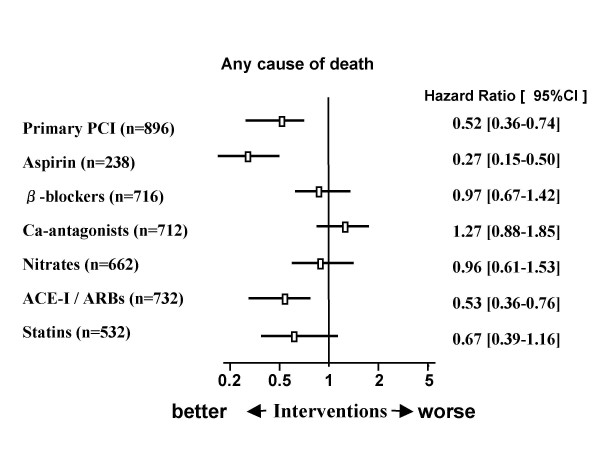
**Hazard ratio estimates and 95% CIs for all-cause mortality associated with therapeutic modalities on the long-term prognosis in the diabetic patients with AMI**.

To elucidate factors with an effect on the long-term prognosis of the diabetic patients with AMI, propensity scores were calculated. Diabetic patients with AMI were divided into two groups according to use or non-use of each therapy, and patients with matched propensity scores were extracted to examine associations with life expectancy. Use of an ACEI or ARB at discharge was significantly related to a better long-term prognosis for diabetic patients with AMI (0.53 [0.36-0.76], p = 0.001). Similarly, use of aspirin at discharge was significantly associated with a good long-term prognosis (0.27 [0.15-0.50], p < 0.0001). In contrast, use of beta-blockers (0.97 [0.67-1.42], p = 0.889), calcium antagonists (1.27 [0.88-1.85], p = 0.205), and nitrates (0.96 [0.61-1.53], p = 0.874) was not associated with prognosis. Doses of statins based on the standard, approved Japanese doses were associated with improved prognosis in AMI patients with diabetes, but not significantly (0.67 [0.39-1.06], p = 0.11). On the other hand, a significant association was found between successful PCI during the acute phase and a favorable long-term prognosis (0.52 [0.36-0.74], p = 0.02; Figure 3).

## Discussion

Observation of the long-term prognosis of AMI patients has shown that acute revascularization and treatment with ACEIs, ARBs, and aspirin are likely to improve life expectancy, even in diabetic patients with myocardial infarction.

An increase in diabetes mellitus, which is associated with global adoption of a Western lifestyle, has become a worldwide problem[9]. The prevalence of coronary artery disease in diabetic patients is reported to be two to four times higher than in non-diabetic patients[10]. The effect of diabetes on secondary prevention in AMI patients in the contemporary acute revascularization era is unclear. Although the in-hospital survival rate of AMI patients with diabetes is comparable to that of non-diabetic AMI patients, diabetic patients with AMI were found to have a higher long-term death rate and a significantly higher incidence of cardiovascular events than non-diabetic patients with AMI.

Early revascularization of the culprit vessel is recognized as a particularly important prognostic factor in patients with AMI. However, the coronary artery disease of diabetic patients tend to be diffuse and involve multiple vessels, as well as being associated with calcification and coronary artery remodeling, so that vessel diameters are reduced, and achieving revascularization or reperfusion is often quite difficult[11]. Diabetes mellitus remains an independent predictor of adverse events after PCI despite advances in interventional techniques and equipment, as well as use of adjunctive pharmacotherapy. Indeed, diabetes mellitus was associated with adverse events after PCI in a recent clinical trial [12]. However, the investigators also demonstrated that the effect of diabetes on angiographic restenosis appeared to be less striking than estimated previously. Furthermore, hyperglycemia enhances platelet aggregation and smooth muscle cell proliferation, so that restenosis is more likely to occur. The BARI study was conducted in diabetic patients with multi-vessel coronary artery disease who received PCI or CABG. Long-term follow-up of these patients revealed that there was a significantly higher incidence of cardiac death among patients treated with PCI than among patients receiving CABG[13]. However, a number of important devices have been developed since the BARI study was conducted. This means that the results of PCI have improved dramatically, so that the difference in outcome between CABG and PCI has decreased. In fact, some studies have shown a better long-term prognosis with PCI than CABG, since it can shorten the time from the onset of stenosis until reperfusion in seriously ill patients with cardiogenic shock[14].

The efficacy of aspirin has been demonstrated by a number of studies conducted in Western countries[15,16]. In recent randomized trials in subjects with diabetes[17,18], the investigators failed to demonstrate that the use of aspirin reduced the risk of cardiovascular events as primary prevention. In the present study, use of aspirin for secondary prevention was shown to be effective for diabetic patients with AMI. Large-scale, randomized, controlled studies will be necessary to verify the efficacy of aspirin in diabetic patients with AMI.

Diabetic patients with AMI showed an improved long-term prognosis when they were treated with statins[19] and beta-blockers[20]. Although we could not confirm such a beneficial effect of statins and beta-blockers for diabetic patients with AMI in the present study, statins showed a non-significant tendency to be effective. This difference in outcomes may be explained by racial differences between Caucasians and Japanese, differences in the pathology of hypercholesterolemia and hypertension, a higher prevalence of coronary vasospasm among Japanese patients[21], the lack of strict blood pressure and lipid level targets in this observational study, and the use of lower doses of these drugs in Japan compared with those in clinical trials conducted in Europe and the USA[22].

The present study demonstrated that there is a significant relationship between treatment with ACEIs and ARBs and a favorable long-term prognosis. These drugs were reported to block neurohumoral factors and to achieve secondary prevention by stabilizing arteriosclerotic lesions[23,24]. The importance of the anti-inflammatory effect of ARBs has also been pointed out, and this is likely to have some effect on diabetic microangiopathy, in particular.

### Study limitations

There are certain limitations in this retrospective analysis of data from a prospective cohort study. The major limitation of the current study is that it is based on a data-driven post hoc analysis of a cohort study. Therefore, therapeutic modalities were not allocated randomly. Although the analysis of the effects of each therapeutic modality on adverse event rates was performed with the powerful propensity score-matching technique, this control was limited to variables for which data were available. In the present study, the percentage of patients who were treated with ACEIs or ARBs was higher than the percentage who were treated with beta-blockers or statins. For this reason, no significant differences were obtained by comparison, which is inevitable in an observational study. Furthermore, this study is not a clinical trial that targeted lipid-lowering. Consequently, participants were treated with standard, Japanese-approved doses of statins. Although statin therapy showed a tendency to improve the prognosis of diabetic patients, a significant improvement was not shown in the present study. We investigated the prognosis of diabetic patients with AMI but did not assess other coronary risk factors that overlap with the components of the metabolic syndrome in this analysis. As the HIJAMI was a database constructed from cardiologists' perspective, it includes no details on diabetic complications, diabetes duration, and type of diabetes. As the epidemiology of cardiovascular disease in Japan is substantially different from that of non-Japanese, one cannot simply assume that the results of the present study can be extrapolated to non-Japanese populations. In the future, randomized, controlled studies should be conducted to elucidate the association of each risk factor with the prognosis of AMI patients with diabetes.

## Conclusion

Although a significant relationship between the presence of diabetes mellitus and in-hospital survival following AMI was not observed in the contemporary acute revascularization era, there was a significant association between diabetes and subsequent adverse events. Among diabetic patients with AMI, early PCI and treatment with aspirin, ACEIs, or ARBs were significantly associated with lower long-term death rates. Thus, in diabetic patients with AMI, acute revascularization should be attempted, as well as treatment with aspirin and ACEIs or ARBs.

## Competing interests

The authors declare that they have no competing interests.

## Authors' contributions

AyTa participated in its design, HO conceived of the study, and participated in its design and coordination, YE participated in clinical follow-up of patients, FM was a member of executive committee, JY performed the enrolment of patients, AtTak participated in clinical follow-up of patients, RK performed the statistical analysis, TS was a member of executive committee, HK was chair of HIJAMI investigators, NH participated in its design and coordination. All authors read and approved the final manuscript.
